# Definitive Radiotherapy for Locally Advanced Adenoid Cystic Carcinoma of the Maxillary Sinus: A Case Report

**DOI:** 10.7759/cureus.111958

**Published:** 2026-07-02

**Authors:** Chakib Fatima Zahra, Lahlou Imane, Karima Nouni, Amine Lachgar, Hanane Elkacemi, Tayeb Kebdani, Khalid Hassouni

**Affiliations:** 1 Radiation Oncology, National Institute of Oncology, Rabat, MAR

**Keywords:** adenoid cystic carcinoma, head and neck cancer, maxillary sinus, radiotherapy, vmat

## Abstract

Adenoid cystic carcinoma (ACC) is a rare malignant tumor of the salivary glands, characterized by slow growth, marked local aggressiveness, and a strong tendency for perineural invasion. Due to nonspecific symptoms, diagnosis is often delayed, and tumors are frequently locally advanced at presentation. While surgery is the standard treatment for resectable disease, definitive radiotherapy is a valid alternative for inoperable cases. We report a case of ACC of the maxillary sinus, staged T4bN2bM0, treated with definitive volumetric modulated arc therapy (VMAT) radiotherapy, with sustained locoregional control at one year. A 56-year-old chronic smoker was diagnosed with ACC of the right maxillary sinus, staged T4bN2bM0, and considered inoperable. The patient was treated with definitive radiotherapy (69.96 Gy; VMAT technique). A partial response was observed on early post-treatment assessment, with sustained locoregional control after one year of follow-up. Definitive radiotherapy can provide effective locoregional control in inoperable cases of ACC, although long-term follow-up remains essential because of the risk of late recurrence.

## Introduction

Adenoid cystic carcinoma (ACC) is a rare tumor accounting for approximately 1% of head and neck cancers. It is characterized by slow growth, prominent perineural invasion, and an insidious clinical course, often leading to delayed diagnosis. At presentation, tumors are frequently locally advanced because of asymptomatic progression and silent nerve infiltration. Maxillary sinus involvement is exceptional, and inoperable cases require effective nonsurgical strategies. We report a case of locally advanced ACC of the maxillary sinus treated with definitive volumetric modulated arc therapy (VMAT) radiotherapy, achieving sustained locoregional control at one year [1-3].

## Case presentation

A 56-year-old man with a history of chronic smoking presented on September 2, 2024, with recurrent epistaxis and right-sided facial pain of three months' duration.

As part of the initial diagnostic workup, a bucco-dental examination performed on the same day revealed poor oral hygiene, absence of trismus, and a 4-cm exophytic ulcerated lesion occupying the entire right hard palate. In addition, several palpable jugular chain lymph nodes were noted.

Maxillofacial CT imaging demonstrated a locally advanced tumor involving the right alveolar bone, measuring 57 × 77 × 50 mm, with associated lytic destruction of the hard palate.

Four months after symptom onset, a biopsy of the oral cavity lesion was performed. Histopathological examination revealed a poorly differentiated carcinoma, prompting immunohistochemical (IHC) analysis for definitive characterization.

Subsequently, an IHC completed shortly thereafter confirmed the diagnosis of ACC (Figure 1).

**Figure 1:**
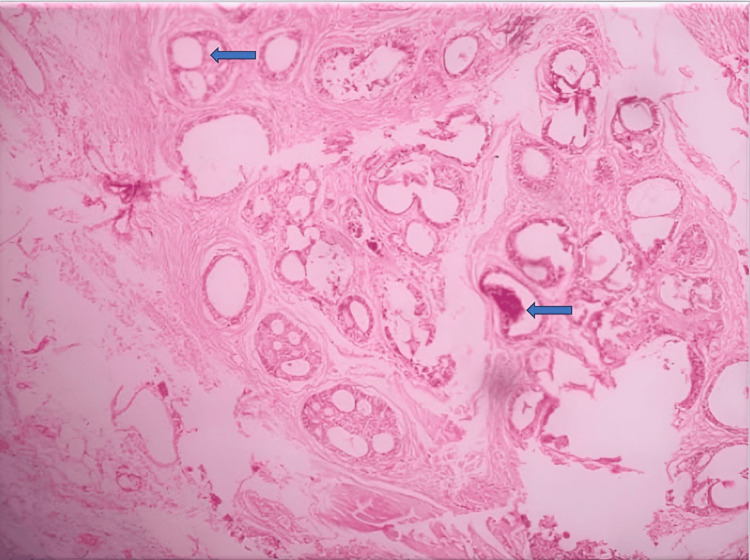
Photomicrograph (H&E stain, ×100) of the maxillary sinus tumor revealing numerous pseudocystic spaces of varying sizes lined by cuboidal cells with scant cytoplasm and oval nuclei. Eosinophilic material is present within the lumina, consistent with ACC ACC, adenoid cystic carcinoma; H&E, hematoxylin and eosin

Following CT imaging, biopsy, and a negative metastatic workup by thoraco-abdomino-pelvic CT, facial MRI confirmed a large right maxillary sinus tumor with locoregional extension, staged as T4bN2bM0 (Figure 2). After a multidisciplinary tumor board discussion, the lesion was deemed inoperable.

**Figure 2:**
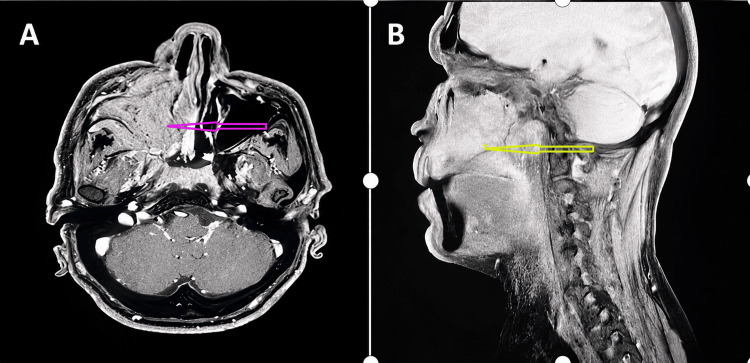
Pretreatment contrast-enhanced MRI (T1-weighted post-gadolinium) of the right maxillary sinus ACC demonstrating extensive locoregional invasion (A) Axial T1-weighted post-gadolinium MRI showing a large, heterogeneous, enhancing mass occupying the right maxillary sinus with extension to adjacent structures (purple arrow). (B) Sagittal T1-weighted post-gadolinium MRI demonstrating locoregional tumor extension toward the skull base and surrounding soft tissues (yellow arrow). ACC, adenoid cystic carcinoma

The patient received definitive radiotherapy using the VMAT technique, delivering a total dose of 69.96 Gy in 33 fractions (Figure 3).

**Figure 3:**
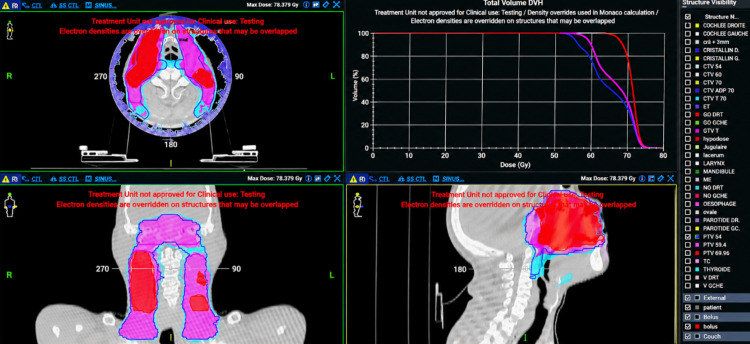
VMAT treatment planning with dose distribution and dose-volume histogram for head and neck radiotherapy The DVH demonstrates dose conformity and target coverage across multiple PTVs: PTV 54 (blue contour): Planning target volume receiving 54 Gy; PTV 59.4 (pink contour): Planning target volume receiving 59.4 Gy; PTV 69.96 (red contour): Planning target volume receiving 69.96 Gy. VMAT, volumetric modulated arc therapy; DVH, dose-volume histogram; PTVs, planning target volumes

Early post-treatment evaluation with facial MRI (Figure 4) showed a partial response with significant tumor volume reduction and clinical improvement.

**Figure 4:**
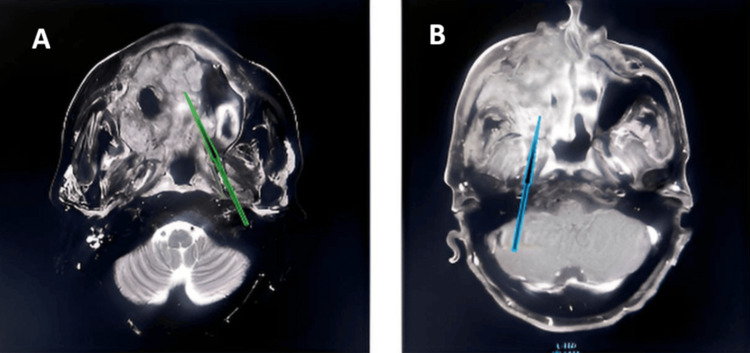
One-year post-treatment contrast-enhanced MRI (T1-weighted post-gadolinium) following definitive VMAT radiotherapy (69.96 Gy), demonstrating a partial radiologic response with marked reduction in tumor volume compared with baseline imaging (A) Axial T1-weighted post-gadolinium MRI showing a residual enhancing lesion within the right maxillary sinus, with substantial reduction in tumor volume compared with pretreatment imaging (green arrow). (B) Axial T1-weighted post-gadolinium MRI at a different anatomic level demonstrating post-treatment changes and residual soft-tissue enhancement within the treated region (blue arrow). VMAT, volumetric modulated arc therapy

After one year of regular follow-up, the patient maintained stable locoregional disease control with a persistent partial response and no evidence of progression.

## Discussion

ACC of the maxillary sinus is a rare and particularly challenging entity due to its late diagnosis, microscopic perineural invasion, and relative radioresistance. Perineural spread represents a major pathway for local recurrence and explains why tumors are often extensive at presentation despite mild initial symptoms [1,2]. The insidious clinical course frequently leads to diagnostic delay, with patients presenting with advanced-stage disease involving adjacent structures such as the orbit, skull base, or infratemporal fossa [3].

In the present case, the tumor was classified as T4bN2bM0 and deemed unresectable after multidisciplinary tumor board discussion. The patient received definitive radiotherapy using VMAT to a total dose of 69.96 Gy in 33 fractions. Early post-treatment imaging demonstrated a partial response, and locoregional control was sustained at one year of follow-up.

The standard of care for resectable ACC is wide surgical excision with negative margins followed by adjuvant radiotherapy [4,5]. However, for locally advanced unresectable tumors, definitive radiotherapy is a well-established alternative [6,7]. Current National Comprehensive Cancer Network (NCCN) guidelines (2025) recommend high radiation doses of at least 66-70 Gy in this setting to optimize locoregional control [8]. The role of radiotherapy in malignant salivary gland tumors has also been well documented [9]. The dose delivered to our patient (69.96 Gy) is consistent with these recommendations.

Modern conformal radiotherapy techniques, particularly intensity-modulated radiotherapy (IMRT) and VMAT, have significantly improved the therapeutic ratio by allowing dose escalation while sparing organs at risk. Several studies have demonstrated that VMAT provides better dose conformity and a higher conformity index than IMRT, particularly for sinonasal tumors, reducing doses to critical structures such as the spinal cord, brainstem, optic nerves, and contralateral retina [5,10,11]. In our patient, VMAT enabled safe delivery of ablative doses without acute grade 3 or higher toxicity.

Despite these advances, ACC remains relatively radioresistant. Tumor response is often slow and incomplete, and complete responses are rare in advanced disease. Published series reports that five-year local control after definitive radiotherapy ranges from 40% to 70%, with incomplete responses being common [4,6]. Nevertheless, a durable partial response can be a clinically meaningful endpoint, as it may translate into prolonged locoregional control and symptom relief. Our patient achieved a sustained partial response at one year, which is consistent with the literature [6,11].

One of the most challenging aspects of ACC is its propensity for late recurrence. Amit et al. (2014) reported that up to 30% of recurrences occur more than 10 years after initial treatment, underscoring the need for prolonged surveillance [3]. Distant metastases, most commonly to the lung, liver, and bone, can also develop decades after locoregional control [10]. Therefore, our patient will require annual imaging follow-up, including sinus MRI and chest CT, for at least 10 years.

The role of systemic therapy in unresectable locally advanced ACC remains investigational. Some phase II trials have explored tyrosine kinase inhibitors such as lenvatinib or apatinib in recurrent or metastatic ACC, but their use as first-line treatment is not established [10-12]. Immunotherapy with anti-PD-1 agents has shown modest activity in salivary gland tumors, except in those with a high tumor mutational burden [10]. In our patient, concurrent systemic therapy was not administered, and definitive radiotherapy alone was sufficient to achieve locoregional control at one year.

Our case has several limitations inherent to a single case report. These include the lack of histologic confirmation of response, the relatively short follow-up duration (one year), and the absence of correlation with prognostic biomarkers such as MYB-NFIB gene fusion or MYB overexpression [8]. Additionally, we cannot exclude the possibility of ultra-late locoregional recurrence or future distant metastasis. Nevertheless, this report illustrates that high-dose definitive VMAT radiotherapy is feasible and effective in achieving early locoregional control in a patient with T4bN2bM0 ACC of the maxillary sinus.

## Conclusions

ACC of the maxillary sinus, although rare, represents a major therapeutic challenge due to its late diagnosis, perineural invasion, and relative radioresistance. In locally advanced unresectable cases (T4b), definitive radiotherapy using modern techniques such as VMAT allows delivery of ablative doses (69.96 Gy) while sparing organs at risk.

At the latest follow-up, the patient remained alive with a sustained partial response, marked tumor volume reduction, and no evidence of locoregional progression one year after definitive VMAT radiotherapy, supporting this approach as a valid alternative when surgery is not feasible. Given the risk of very late recurrences, prolonged follow-up beyond ten years is essential, combining locoregional imaging and screening for distant metastases. Prospective multicenter studies are needed to define predictive factors of response and clarify the potential role of targeted therapies and immunotherapy in combination with radiotherapy for these rare tumors.
